# A New Method for Ultrasound Detection of Interfacial Position in Gas-Liquid Two-Phase Flow

**DOI:** 10.3390/s140509093

**Published:** 2014-05-22

**Authors:** Fábio Rizental Coutinho, César Yutaka Ofuchi, Lúcia Valéria Ramos de Arruda, Flávio Neves Jr., Rigoberto E. M. Morales

**Affiliations:** 1 Department of Electronic Engineering, Federal University of Technology—Paraná (UTFPR), Campus Toledo-PR, 85902-490, Brazil; 2 Graduate School on Electrical Engineering and Applied Computer Science, Federal University of Technology—Paraná (UTFPR), Campus Curitiba-PR, 80230-901, Brazil; E-Mails: ofuchi@utfpr.edu.br (C.Y.O.); lvrarruda@utfpr.edu.br (L.V.R.A.); neves@utfpr.edu.br (F.N.J.); rmorales@utfpr.edu.br (R.E.M.M.)

**Keywords:** ultrasound, Doppler method, multiphase flow, gas liquid two-phase flow

## Abstract

Ultrasonic measurement techniques for velocity estimation are currently widely used in fluid flow studies and applications. An accurate determination of interfacial position in gas-liquid two-phase flows is still an open problem. The quality of this information directly reflects on the accuracy of void fraction measurement, and it provides a means of discriminating velocity information of both phases. The algorithm known as Velocity Matched Spectrum (VM Spectrum) is a velocity estimator that stands out from other methods by returning a spectrum of velocities for each interrogated volume sample. Interface detection of free-rising bubbles in quiescent liquid presents some difficulties for interface detection due to abrupt changes in interface inclination. In this work a method based on velocity spectrum curve shape is used to generate a spatial-temporal mapping, which, after spatial filtering, yields an accurate contour of the air-water interface. It is shown that the proposed technique yields a RMS error between 1.71 and 3.39 and a probability of detection failure and false detection between 0.89% and 11.9% in determining the spatial-temporal gas-liquid interface position in the flow of free rising bubbles in stagnant liquid. This result is valid for both free path and with transducer emitting through a metallic plate or a Plexiglas pipe.

## 1. Introduction

Interfacial behavior plays a fundamental role in gas-liquid flows because it directly reflects the combination of the phase flow rates. The estimation of interface position changes over time provides information for measuring the mean void fraction in two-phase pipe flows. A technique combining high speed footage and image processing can achieve high spatial and moderate temporal accuracy in interface detection [1]. However, high speed footage is guaranteed only if both pipe and fluids are optically transparent, a restriction that severely limits the application of this technique. Impedance probes arranged in a wire-mesh configuration [2] can be applied to a much larger number of fluid types than optical techniques, achieving high spatial and temporal resolution. Yet, their applications are restricted to flow visualization and validation purposes since it disturbs the flow due to its intrusive nature. X-rays and radiation-based techniques [3,4] overcome most of the drawbacks in the aforementioned methodologies. On the other hand, environmental issues, high cost and the difficult of handling nuclear sources prevent a large acceptance by industries.

In this context, the use of techniques that explore the interaction of ultrasonic waves with matter can be very promising. As a mechanical wave, ultrasound propagates smoothly through solid and liquid media, allowing transducer installation externally to the pipeline. Therefore, it is a non-intrusive and non-invasive measurement technique. It can also be considered as a low cost and easy handling relatively to other techniques, chiefly nuclear based ones. Besides, ultrasound techniques can be applied to several types of liquids, including opaque ones such as heavy oil.

Pulsed-wave ultrasound systems for velocity estimation are now commonly used in fluid mechanics. However, when they are applied to a two-phase flow, phase separation of velocity measured is still an open problem. Therefore, a lot of research effort has been dedicated to obtain a framework that can estimate velocity profile with additional information on which phase it came from. Aritomi *et al.* [5] and Suzuki *et al.* [6] developed a method to distinguish phase velocity by applying a statistical method in velocity data measured in a countercurrent flow (liquid downward an bubbles upward). Murakawa *et al.* [7,8] used a unique transducer with two elements of different sizes emitting in two separate frequencies. The difference in element size and pulse frequency was selected in order for one to be more sensitive to liquid velocity whereas the other more sensitive to bubble velocity. Murakawa *et al.* [9] also proposed that the liquid-gas interface can be detected by the phase change that occurs in pulse due to acoustic impedance mismatch between liquid-gas interface. An alternative velocity estimation algorithm denoted Velocity Matched Spectrum (VMS), proposed by Torp [10] distinguishes from conventional estimators. Instead of only returning the estimated velocity for each interrogated volume sample, it also computes a velocity spectrum which the corresponding amplitude data are related to the scatterers echo energy. The cost of outputting more data for each estimated velocity is an increase in the computational charge necessary to perform the estimation. However, this new information offers the potential to develop a new technique for phase separation in ultrasonic velocity estimation applied to fluid flow.

An ultrasonic transducer positioned almost perpendicularly to the pipe, with a small deviation angle of 5 to 7 degrees, can obtain velocity and receive echoes from gas-liquid interface. Thus the interfacial position can be easily detected by searching for high amplitude echoes that exceeds an established threshold. However, this echo intensity technique fails in presence of low signal-to-noise ratio, usually caused by the coupling between transducer and medium or by liquid with high attenuation of ultrasound waves. Sudden changes in interface inclination angle can also result in failure (no detection) since no echo is received by the transducer [11]. Free-rising bubbles in a quiescent liquid present high velocity distribution around bubble interface, and since liquid velocity is very low far from the bubble rise path, the interfacial position can be easily detected by searching for high velocity data points due to bubble wake velocity pattern [12]. In [13], a similar framework was used to measure position and velocity of single bubble rising in a liquid metal. The ultrasonic transducer was directed vertically along bubble path. Nevertheless, these frameworks are restricted to a sufficient particle concentration and are subjected to velocity estimation fluctuations due to the stagnant liquid which demands the use of spatial filtering for reliable detection.

In this work, we extract several shape parameters from velocity spectrum data to generate a spatial-temporal map to be used for phase identification purposes, through utilization of VMS algorithm, in a free-rising bubble flow. This two-dimensional map is considered as an image. The interfacial position is obtained by applying filtering and edge detection algorithm on this image. The method is denoted “velocity spectrum technique”, since a velocity spectrum curve is used to support computation and analysis. The result of application of this new technique is compared to the corresponding intensity echo method showing that it greatly improves SNR, consequently resulting in a more reliable performance. Since detection is based solely on the echo energy from air-water interface it is not limited to interface scale and can be applied even when particles are not present. Estimated velocity data can be used to improve detection accuracy performance but are not mandatory for detection. Experimental results show that the proposed technique results in an accurate profile of the air-water interface even *in situ*ations where transducer is obstructed by either a metallic plate or a Plexiglas pipe.

## 2. Foundation of the Velocity Spectrum Technique

This section covers the theoretical concepts that support the proposed Velocity Spectrum (VS) technique. It begins by reviewing the velocity estimation method used to obtain the velocity spectrum, followed by an explanation of the spectrum relationship with the liquid-gas interface and concluding with the methodology used to quantify shape parameters extracted from the estimated velocity spectrum. It should be noted that VS technique requires a final step that involves spatial filtering and depends on the applied flow. Because of this dependency, this step will be explained in the next section after the description of the experimental configuration.

### 2.1. Velocity Estimation by Velocity Matched Spectrum Technique

**Figure 1.**
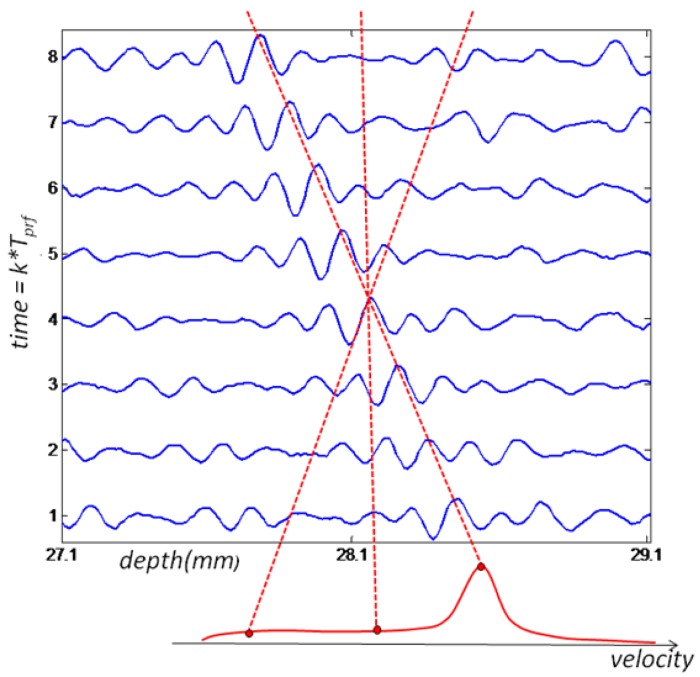
Example of 8 ultrasound emissions. Velocity spectrum is obtained by calculating 1D FFT along skewed lines in depth/time plane.

The Velocity Matched Spectrum (VMS) algorithm differs from conventional Doppler estimators because it requires more than one sample over a certain depth range. Therefore ultrasound signals received from multiple emissions are treated as 2D vectors (Figure 1) where the vertical axis is associated with the *k*th emission, occurring every *T_prf_* seconds, and the horizontal axis is related to the pulse time of flight, *t*, corresponding to a depth range, *d* = *ct*/2, where *c* is the speed of sound. Due to the relative movement of the acoustic reflector from the transducer, a time shift in received echoes will be observed at each emission. This delay is correlated with the scatterers velocity. Moreover, the entire depth is divided in range gates, according to the desired space resolution and it cannot be less than the minimum axial resolution given by the transducer used. Only a number of emissions are considered depending on the target time resolution. Thus velocity estimation is done for each data matrix, *x* (*t*, *k*) for a certain range and number of emissions.

**Figure 2.**
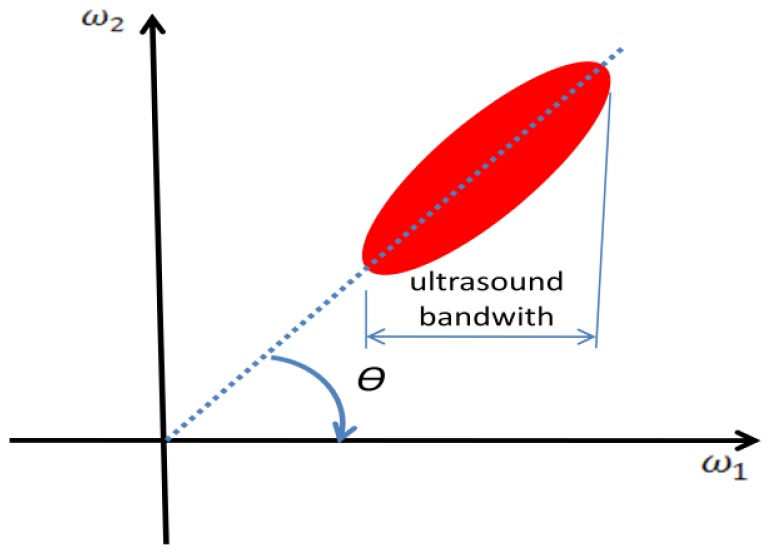
Data mapping in two-dimensional Fourier space showing ideal response for a pulsed Doppler ultrasound signal (complex envelope).

Since large quantities of acoustic reflectors are artificially inserted in the flow, the received data matrix will comprise multiple reflections, as shown in Figure 1. Thus the signal received is treated as a stochastic process and can be modeled as a complex Gaussian process [14]. Such process is completely characterized by its autocorrelation and the power density spectrum defined as

(1) $$R_{x}\left(\tau ,m\right)=E\left[x{\left(t,k\right)}^{*}x\left(t+\tau ,k+m\right)\right]$$

(2) $$G_{x}\left(\omega_{1},\omega_{2}\right)=\int^{}\int^{}R_{x}\left(\tau ,m\right)e^{-i\omega_{1}\tau }e^{-i\omega_{2}mT_{\text{prf}}}d\tau dm$$

where * stands for complex conjugate operator; *x*(*t*, *k*) is the complex pre-envelope of the receiver signal, assumed as being time stationary and a continuous function, τ and *m* are the time-lags in pulse time of flight, *t*, and the *k*th emission, *k*. *G_x_*(*ω*_1_, *ω*_2_) is only the nonaliased part of the power spectrum, where *ω*_1_ can be identified with the frequency of the received echoes and *ω*_2_ can be identified with Doppler shift. The autocorrelation function is described by the point scatterer response, *s*(*t*) , and transversal beam sensitivity function, *b*(*d*), where *d* is the distance from ultrasonic beam center axis, as [14]

(3) $$\begin{array}{c} R_{x}\left(\tau ,m\right)=s_{2}\left(\tau -mt_{s}\right)b_{2}\left(v_{t}T_{\text{prf}}m\right)\\ t_{s}=-\frac{2v_{r}}{c}T_{\text{prf}} \end{array}$$

here subscripts 2 at *s*_2_ and *b*_2_indicate short notations for the autocorrelation operator applied to *s* and *b*. The time shift due to particle relative motion in radial direction is described by *t_s_*, where *v_r_* and *v_t_* are the velocity components in the radial axis of ultrasonic beam and transversal to ultrasonic beam, respectively. Due to convention, *t_s_* has a negative sign to indicate that the particles are moving away from the transducer. Substituting Equation (3) in Equation (2), the nonaliased part of spectrum will be

(4) $$G_{x}\left(\omega_{1},\omega_{2}\right)={\left|S\left(\omega_{1}\right)\right|}^{2}{\left|B\left[\frac{1}{v_{t}}\left(\omega_{2}-\frac{2v_{r}}{c}\omega_{1}\right)\right]\right|}^{2}$$

where functions *S*(*ω*_1_) and *B*(*ω*_2_) are the Fourier transforms of *s* and *b* respectively. The argument of function *B* is zero along the straight line with slope in the (*ω*_1_, *ω*_2_) plane, which represents the time shift, *t_s_*, between emissions. The spectral energy of *B* will be concentrated along this line due to low-pass characteristic of this function (Figure 2). The ultrasound pulse bandwidth is given by frequency response of *S*(*ω*_1_), which limits the spectral line of *B* to the bandwidth of the ultrasound pulse in *ω*_1_ direction (Figure 2). By analyzing the major ellipse axis orientation from Figure 2, the angle, θ, will be related to the argument of *B* by the relation

(5) $$\tan\left(\theta \right)=\frac{2}{c}v_{r}$$

The 2D power spectrum can be estimated through 2D FFT technique using as argument the 2D data array of complex pre-envelopes multiplied by a 2D Hamming window, defined as *w*(*x*)*w*(*t*), where *w*(*x*) is the one-dimensional Hamming window. The velocity spectrum is built by integrating the estimated 2D spectrum along straight lines through the origin, such as the approach adopted by [15,16]. Another method for building the velocity spectrum was proposed in [14], and it involves calculating the 1D Fourier transform of the signal along skewed lines in the depth/time plane (Figure 1), where the slope is chosen to follow the movement of the scatterers from pulse to pulse for each considered velocity. Both algorithms are equivalent, being the latter less computationally intensive.

The velocity matched spectrum technique differs from the others by the amount of information that can be obtained by carefully analyzing the velocity spectrum. In conventional estimators only the mean velocity of the sample volume is returned. The VMS method provides not only the velocity data, but also its correspondent amplitude distribution of the velocity spectrum. The amount of returned data comes with the cost of a computationally intensive algorithm, mainly because of FFT evaluations. The peak of the velocity spectrum indicates the most likely angle of the major ellipse axis which by its turn is a high quality measure of the scatterers velocity [16].

**Figure 3.**
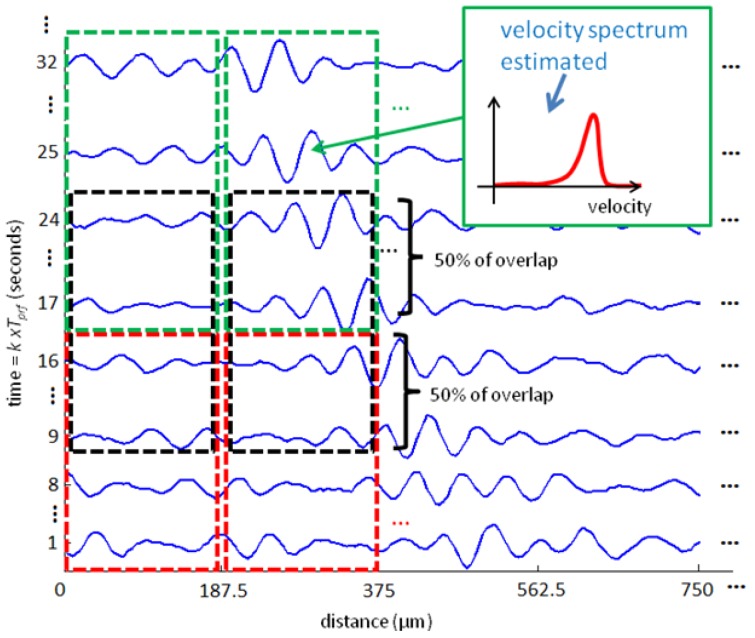
Example of data matrix subdivision. Each input matrix (dashed boxes) comprises 187.5 μm of range samples and 16 × T_prf_ seconds of time samples or 16 ultrasound emissions. Velocity spectrum is estimated for each input matrix. Overlap in time dimension of input data can be used (black dashed box), but with no gain in time resolution.

To implement the VMS method, the two-dimensional data shown in Figure 1 is sampled in depth dimension, resulting in a discrete range-time matrix. This matrix is then subdivided in smaller matrices that will serve as input for velocity estimation (Figure 3). The matrix size chosen will result in the range and time resolution of the velocity measured.

It should be noted that VMS method performance can be degraded if there are multiple targets, with different velocities, mainly bubbles, showing in the distance-time map used as input to the velocity estimation algorithm. In this case, the velocity spectrum will present multiple peaks which will compromise velocity estimation. However this situation can be avoided by estimating the velocity spectrum for very small range and very short time period, Figure 3 is an example. The choice of resolution implicates in a priori knowledge about the flow. For example, the time resolution can be chosen as the largest velocity possible in the flow and the range resolution must be the size of the smallest bubble that can occur.

In this work it is shown that the shape of velocity spectrum is related to the presence of the liquid-gas interface, therefore, it is used to an accurate and reliable evaluation of the spatial-temporal position of the interface.

### 2.2. Liquid-Gas Interface Detection

When the VM spectrum algorithm is applied to a gas-liquid flow, and since ultrasound waves almost reflect totally at the interface, echoes received from the air-water boundary will have high amplitude and therefore high energy velocity spectrum. Hence the interface position can be resolved by analyzing the velocity spectrum data. That is the foundation of the proposed technique.

To reliably detect interface position, a technique based on velocity spectrum called Velocity Spectrum technique (or VS technique for short) was developed. It consists of extracting a shape parameter from velocity spectrum which will form a spatiotemporal distribution. This two-dimensional data can be viewed as an image and bubble interfaces will appear as high intensity pixels. By applying an edge detection algorithm, interface pixels can be detected and interfacial spatiotemporal position can be easily evaluated.

Since there are several edge detection gradient-based methods, the accuracy performance is evaluated by a set of edge algorithms, by applying them to a free-rising bubble flow experiment, and then selecting the one that gives the best results.

### 2.3. Velocity Spectrum Shape Parameters

The success of the VS technique depends crucially on the input data quality. Since the proposed technique extracts data from the analysis of the velocity spectrum curve and there are a large numbers of parameters that could be obtained from this spectrum, a careful study must be carried out. The main objective is to find a parameter that works as a phase function showing a considerable deviation of values when it is extracted from a gas phase spectrum to the one extracted from a liquid phase spectrum. These contrasting values ease interface identification in the presence of noise.

The *n*-th moment of the velocity spectrum function can be written as

(6) $$M_{n}=\int_{-\infty }^{+\infty }v^{n}f(v)dv$$

which gives a quantitative evaluation of the spectrum shape, where *ν* is the velocity and *f*(*ν*) is the velocity spectrum function. Only the first three moments were chosen because the third and fourth result in symmetry and flatness, and will not be closely related to the energy of the echoes. It was also chosen to study the spectrum peak value and the squared spectrum peak value because they are related to the estimated velocity.

A candidate phase function must have a high signal-to-noise ratio to increase both probability and accuracy for resolving the bubble position. Unfortunately, blind SNR estimation is an open issue in image processing theory. The ratio between maximum and minimum local variances as described in [17] can be used as a rough SNR estimate and it is defined as

(7) $$\text{SNR}=10\log_{10}\left(\frac{\max\left(\delta_{I}^{2}\right)}{\min\left(\delta_{I}^{2}\right)}\right)$$

where $\delta_{I}^{2}$ is the local variance of image *I* at position (*i*, *j*) defined as

(8) $${\delta_{I}}^{2}\left(i,j\right)=\frac{1}{\left(2p+1\right)\left(2q+1\right)}\sum_{k=-p}^{p}\sum_{l=-q}^{q}{\left[I\left(i+k,j+l\right)-\mu_{I}\left(i,j\right)\right]}^{2}$$

where *p* and *q* are the sizes of the local area, *I* is an image formed by the spatiotemporal distribution of the considered shape parameter and μ*_I_* the local mean value which is defined as

(9) $$\mu_{I}=\frac{1}{\left(2p+1\right)\left(2q+1\right)}\sum_{k=-p}^{p}\sum_{l=-q}^{q}I\left(i+k,j+l\right)$$

in this study the local area size was defined as *p* = 2 and *q* = 2, as suggested in [17].

However, absolute values of SNR evaluated by Equation (7) for different shape parameters will not be a good comparison criterion because bulk data (mostly from liquid phase) can vary from one shape parameter to another. To overcome this problem, a relative value took from the division of SNR from liquid + gas by the SNR from only liquid phase through splitting in half (in spatial direction) the spatiotemporal data, *I*, was used. The first part contains only pixels caused by particle echoes and no bubble pixels. The second part consists of bubble plus particles echoes. By dividing the latter part by the former ones, the shape decision rule is defined, namely:

(10) $$\text{SDR}=\frac{\text{SNR}(I(i_{2},j))}{\text{SNR}(I(i_{1},j))},i_{1}=1,2,..{\text{N}}_{1},i_{2}={\text{N}}_{1}+1,\text{N}+2,..\text{N},{\text{N}}_{1}<\text{N and}\;j=1,2,\ldots \text{M}$$

where *I* is an image formed by the spatiotemporal distribution of the considered shape parameter, N is the total number of depth pixels, N_1_ is the total number of pixels that only have particle echoes and M is the total number of temporal pixels. In experimental setup, bubble generation occurs in a controlled region and N_1_ is usually defined as N/2. In Equation (10), if no bubbles are present in the image *I*, SNR from numerator will be ideally equal to SNR from denominator thus SDR will be 1. When bubbles are present in image *I*(*i*_2_, *j*), SNR from numerator will be bigger than SNR in the denominator resulting, ideally, in some SDR value bigger than 1. It should be pointed out that because ultrasound attenuates with distance it is possible to have SDR less than one since it is a division from echoes of a greater distance by echoes near to the transducer. However, values much smaller than one in SDR will indicate that no bubbles are present or that SNR it is too low for detection.

## 3. Free-Rising Bubble Flow

Bubbles rising in a quiescent liquid suffer from a constant change in interfacial slope, due to shape deformations that occur in their wobbling ascension. Moreover if the bubble size has the same order of ultrasound pulse length, the ultrasonic interface detection results in a complex task [11]. In this section, the proposed technique is applied to four different variations of free rising bubbles flow to demonstrate its applicability and reliability. In the first experiment, the methodology used to calculate SDR for all free-rising bubble flow experiments is explained. The last part of VS technique, which is experiment dependent, is also presented in the first experiment and it is valid for all described experiments.

### 3.1. Free Path Experiment

**Figure 4.**
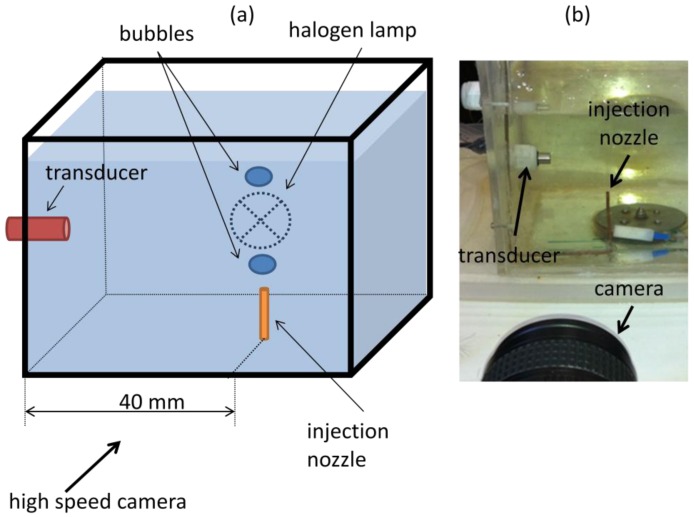
(**a**) Experimental set-up diagram for interface detection of free-rising bubbles in quiescent liquid. (**b**) Photograph of measurement environment from camera point of view.

A water tank was built and filled with mineral water. Powder with diameters from 80 μm to 200 μm (EMS GRILTECH 1A P82), with 1.07 g/cm^3^ was added to the water in the tank to a concentration of 4 g/L. To generate air bubbles, an injection nozzle aligned with the ultrasound transducer was mounted at the bottom of the tank as displayed in Figure 4a. A high speed camera was used to record bubble motion, Figure 4b, with a frame rate of 250 fps and it was positioned to give a 45 μm/pixel resolution. To provide adequate illumination, a 150 W halogen lamp was installed behind the tank, facing the camera.

For all experiments described in this paper, ultrasound transducer was mounted horizontally so as to facilitate assembly into the tank and to eliminate angle uncertainty in case of an oblique position. This horizontal assembly will make the transducer sensible only to bubble horizontal velocity component. As bubble follows helical motion it will present a significant horizontal velocity component. Oblique ultrasound emission should perform similarly if backscattered echoes return for the transducer active diameter.

The transducer used has a central frequency of 8 MHz, which was chosen because it will have a wavelength of 187.5 μm in water. Transducer mechanical design is of a cylindrical shape with overall diameter of 8 mm and 40 mm of length. It has a flat active element with 2.5 mm of diameter at front face. A 1-cycle pulse and 2 kHz of pulse repetition frequency was configured at the ultrasound Pulser.

**Figure 5.**
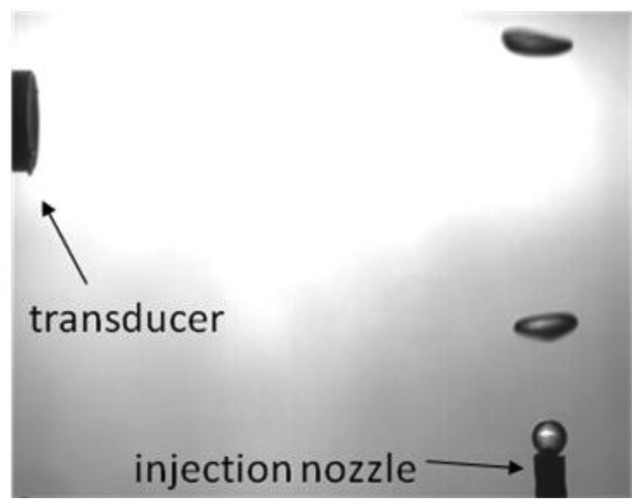
Image sample from high speed filming employed on mean sphere equivalent diameter estimation.

An air pump connected to the injection nozzle was adjusted to generate bubbles with sphere-equivalent diameter from 2.5 mm to 3.5 mm. Image processing of 15 bubble image samples, Figure 5, was done and confirmed a mean sphere-equivalent diameter of 3 mm.

**Figure 6.**
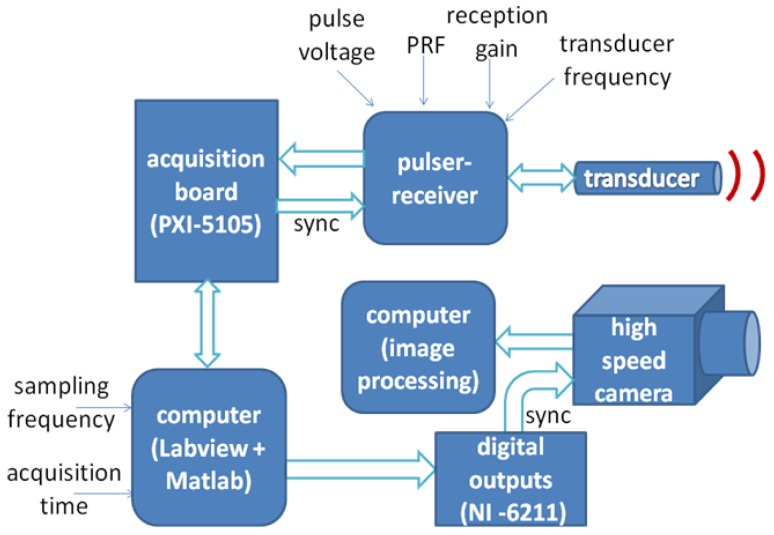
Block diagram of the measurement system developed to implement VMS method.

Due to the lack of specialized equipment that implements the VMS algorithm for application in fluid flow, a measurement system was developed in order to validate the proposed method. The overall system is displayed in Figure 6.

The developed measurement system comprises an ultrasound pulser-receiver, using a commercial equipment from Panametrics, model 5077PR, operating in a pulse-echo mode, 1-cycle pulse, which generates appropriate excitation for an ultrasound transducer and incorporates an analog front-end to amplify or attenuate the received echoes, if desired. The signals are then digitized by a PXI system, model 1001B, equipped with a data acquisition board PXI-5105, which achieves a maximum of 60 MS/s and 12-bits of resolution. A Labview program was developed to control data acquisition performed by the PXI system and to store data. Labview communicates via USB port to a NI-6211 board to generate a start signal (sync) to the high speed camera, which was used for validation purposes. By means of the synchronization signal (sync), the data acquisition starts simultaneously in both ultrasound and camera side. Finally the data stored is processed in a Matlab environment through a script that implements the velocity estimator by VM-Spectrum method.

The measurement system was configured to give a spatiotemporal resolution of 187.5 μm and 8 ms to estimate the instantaneous Doppler velocity and correspondent spectrum amplitude. To equalize the number of points estimated in temporal dimension to the 250 fps from the camera, the input data was overlapped in time axis by a factor of 50% (as shown in Figure 3).

To evaluate the performance of the aforementioned shape parameters, 264 bubbles were injected by the nozzle corresponding to 15 s of data acquisition. Received ultrasound echoes were amplified by 20 dB by manually adjusting the Pulser-Receiver gain parameter.

**Figure 7.**
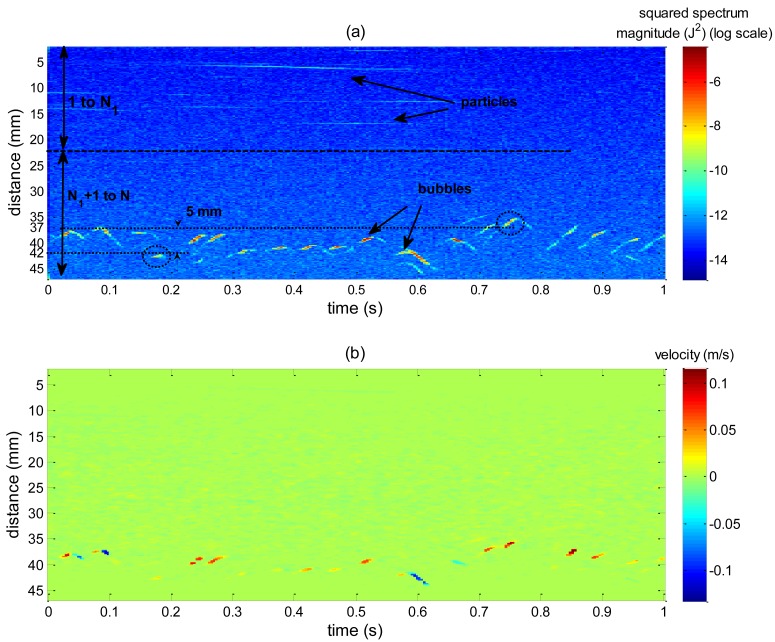
Example of squared spectrum peak distribution and corresponding velocity map. (**a**) The division of the spatiotemporal map in half by spatial dimension for SDR computation is showed for 1 second of acquisition build with squared spectrum peak. (**b**) Velocity distribution where bubbles are characterized by high values of velocity (0.1 m/s and −0.1 m/s) and particles near the transducer that are highlighted in (a) cannot be seen in (b) because they present null velocities.

To apply the VS technique for interface detection it is necessary to build a spatiotemporal map based on a shape parameter obtained from the application of the VMS algorithm. The shape parameter is chosen by computing the SDR rule in Equation (10). The image formed by the spatiotemporal distribution of each candidate to be used for phase identification purposes was split, in spatial dimension, for the SDR computation as shown in Figure 7a. Since bubble generation occurs 40 mm away from the transducer, in the first half (Figure 7a) no bubble will cross the ultrasound path even with a very large wobbling motion, so N_1_ corresponds to half of the maximum acquired distance (47 mm) because between 1 and N_1_ no bubble data will be found.

Table 1 summarizes the results of SDR evaluation. Spectrum mean, (M_1_), is chosen for phase identification purposes, because it presented the higher value, indicating that it has a high SNR ratio among the other shape parameters. The specific value of 1.53 shows that the second part of the distribution of M_1_ (from N_1_ + 1 to N), in which bubbles disturbances are present, has a 53% higher SNR value than the part which comprises only liquid and particles disturbances.

**Figure 8.**
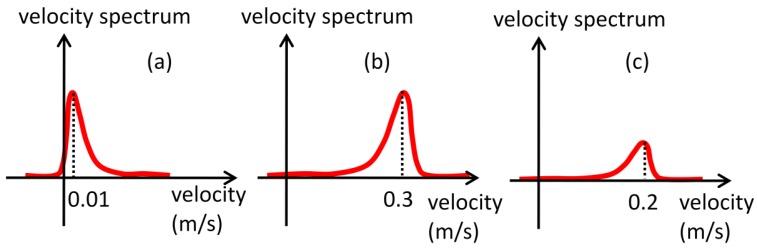
(**a**) Velocity spectrum for particles near the transducer. (**b**) Velocity spectrum for bubbles. (**c**) Velocity spectrum for particles far from transducer and in the bubble raster route.

In free-rising bubbles, particles near the transducer will result in high amplitude echoes. If powder concentration is high this will generate even stronger echoes because of the superposition of backscattered waves. But, since they are far from bubble rise generation, they experience very low velocities, Figure 8a, thus resulting in low M_1_ values. Bubbles far from the transducer will also generate strong echoes because of the acoustic impedance difference and will present high velocity values, Figure 8b, resulting in high M_1_ value. Particles near bubbles that are in the raster path will present high velocity but weak echo energy, Figure 8c, so M_1_ will present moderate values. Therefore, because of this large slip condition, M_1_ will present best SDR results. In low slip condition, M_1_ will not perform equally. Thus, in this situation, particle concentration should be reduced, resulting in weak echoes near the transducer. There are studies showing that concentration of order mg/L can be successfully used without compromising velocity estimation if flow velocity is sufficient high [18]. Spectrum peak and squared spectrum peak parameter are unaffected by velocity difference, because they are not weighted by velocity as the moments. Therefore, it is expected that in low slip conditions they should surpass other parameters for interface detection.

However, due to the high particles concentration used (4 g/L), M_1_ values from regions near the transducer will still be high and can lead to false interface detection. Therefore the task of detecting bubble interface becomes tougher. In such high concentration scenario particle echoes can be eliminated by multiplying the estimated velocity at each spatiotemporal point by the corresponding value of the used shape parameter, in this case M_1_. Figure 7b shows a sample of velocity map obtained for 1 second of acquisition, where velocities were estimated by the peak in spectrum amplitude. Since most of liquid is stagnant, thereby resulting in several velocity points near zero in the bubble generation neighborhood, the velocity will be high as shown in Figure 7b. So this operation clearly functions as a filter emphasizing only points near the liquid-gas interface. This procedure will be referred as velocity filtering. This situation can be avoided if particle concentration can be reduced, resulting in weak echoes (in the experiment a high concentration (4 g/L) was intentionally used to test the technique in the worst-case scenario).

**Figure 9.**
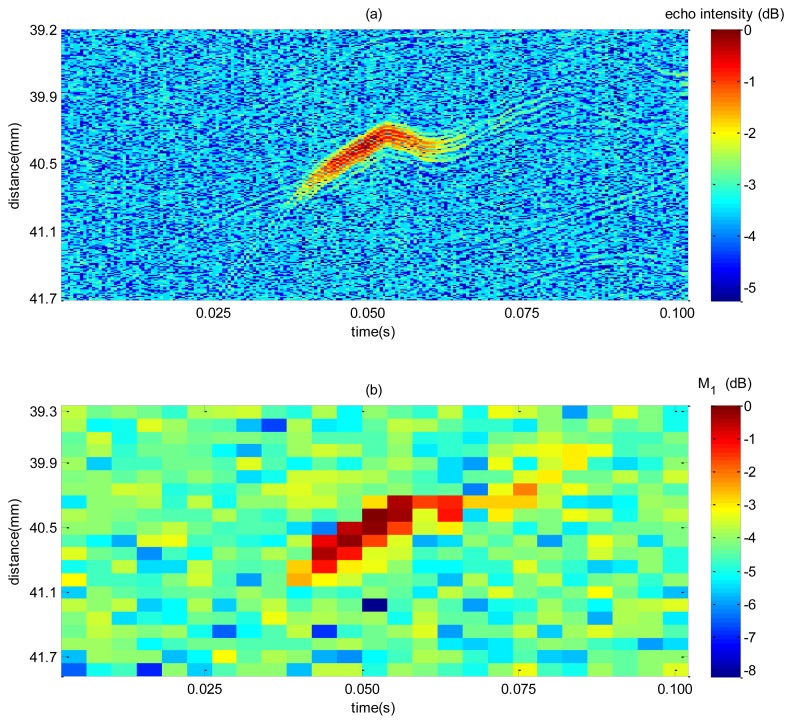
Comparison of bubble disturbance in a map built with M_1_ and with echo intensity. In (**a**) echo intensity spatiotemporal map showing disturbance caused by a bubble pass. (**b**) M_1_ map corresponding to the same time-distance range described in (a).

A sample of the spectrum mean and intensity echo map is illustrated in Figure 9 for a single bubble rising. The magnitude of the maps from Figure 9 was normalized by the highest value measured. Echo intensity image, Figure 9a, gives a higher spatial resolution so that it would be more accurate whenever a bubble is detected. However, in a low SNR condition, selecting a threshold is a difficult task because high threshold can miss out bubbles, and a too low threshold can give false bubble indication. VS technique enhances the bulk to bubble amplitude difference thus making threshold selection easier and giving a more reliable detection at the expense of a decrease in resolution. This can be noticed in Figure 9b where gas-liquid interface is characterized by an amplitude of approximately 4.3 dB higher from bulk (mean bulk is equal to 4.3 dB). In contrast, echo intensity distributions (Figure 9a), a 3.7 dB of difference are achieved (mean bulk is equal to 3.7 dB). Therefore, it is expected that the use of the former technique will be more efficient than the latter as for detecting bubbles.

The first moment distribution after velocity filtering can be treated as an image, thus allowing the use of image processing techniques. For edge detection, the first step is to apply a spatial filter which will emphasize bubble boundary. Since various ways of sharpening spatial filters could be used, several tests were performed so as to choose the appropriate one. The target filters masks selected are listed in Table 2. The analytic method for operator design proposed by Canny [19] was also tested as it is known for its good performance for low SNR images. Filtering was applied to consecutive data blocks comprising 1 second of acquisition.To assess detection accuracy the RMS error in the spatial-temporal position was evaluated for different filter methods, as defined in [11] as

(11) $$E=\sqrt{{\left(\frac{\partial_{x}}{\Delta_{x}}\right)}^{2}+{\left(\frac{\partial_{t}}{\Delta_{t}}\right)}^{2}}$$

where *∂_x_* and *∂_t_* are spatial and temporal error and the denominators Δ*_x_* and Δ*_t_* are the corresponding spatial and temporal resolution configured in the measurement system.

The same experimental setup used for spectrum shape parameter test was also used for this error evaluation with the total of 264 bubble passes. Through high speed camera image processing, the relative position of liquid-gas interface was evaluated and used as a reference value for error estimation.

Bubbles might also pass undetected when bubble passage does not produce a significant peak value. Thus a probability of detection failure (PDF) was computed in order to evaluate this problem. Transducer focal distance is 8.5 mm and diverge half angle 2.2 degrees. Therefore, at 40 mm far from transducer face (where injection nozzle is located), ultrasound beam diameter will be of approximately 5 mm. Analyzing Figure 7a, it can be seen that bubble oscillatory motion can reach as far as 10 mm of amplitude. Considering an axis-symmetric oscillation, the same amplitude value would be observed in transducer lateral axis. The dashed circles in Figure 7a indicate two bubbles that are 5 mm far from each other. Therefore, considering a beam diameter of 5 mm and axis-symmetrical oscillation, it is possible that a bubble can pass undetected by circumventing the ultrasound beam. Thus, bubble trajectories that circumvent the ultrasound beam are not included in the PDF computation. To define whether the bubble circumvented ultrasound beam, the footage was synchronized to the spatiotemporal map measured (echo intensity and shape parameter). By visual analysis, when a bubble was observed by the camera but no variation of values in the corresponding spatiotemporal map was found, the bubble trajectory is considered out of transducer beam and bubble is not considered for probability computation. To double check, the camera images of the circumvented bubbles were also analyzed. Camera was adjusted to give the best focus on bubbles that crosses transducer beam. Therefore, bubbles that circumvented the beam appear out of focus in camera image. This was used as a second confirmation to declare a bubble as a circumvented one. The percentage of circumvented bubbles varied from 1.6% to 5%. It should be noted that the detection failure bubbles can be distinguished from circumvented bubbles by checking spatiotemporal map. Circumvented bubbles do not produce disturbance values that can be noticed by visual analysis of the spatiotemporal map. But, disturbances due to detection failure bubbles can be easily noticed by visual analysis of the spatiotemporal map. However, these disturbance magnitude values are not enough to allow interface detection by VS technique.

**Figure 10.**
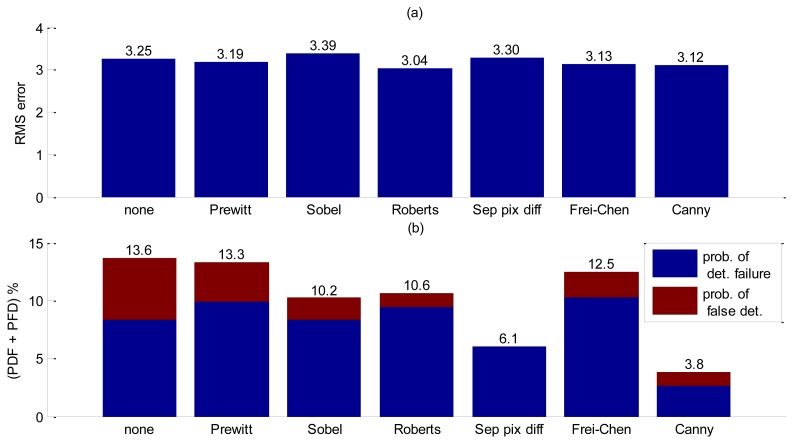
Accuracy performance results for selected spatial filters. (**a**) RMS error results. (**b**) Probability of false detection and detection failure results.

If noise levels in a group of pixels exceed the threshold, false interface detection might happen, therefore a probability of false detection (PFD) was also computed. Since both probabilities affect accuracy, these computations for each filter were added in Figure 10b to allow a fair comparison. The results are summarized in Figure 10a,b.

Analyzing Figure 10b clear the benefit of spatial filtering is clear. Without using spatial filtering, low frequency noise combined with low contrast values between background noise and interface severely increases bubble false detection. Decreasing the threshold so as to reduce PFD will implicate in an increasing probability of detection failure. The Canny filtering has presented the best trade-offs among all other essayed filters for this experiment.

### 3.2. Without Seeding Particles

Based on the fact that the proposed method detects interfaces by analyzing the velocity spectrum energy which depends only on bubble echoes, one would expect that the proposed technique do not demand the presence of tracing powder. Our objective in this study is to verify whether the proposed technique can be applied to that situation or if any modifications should be implemented so as to accurately detect the interface position.

For this condition, the same experimental set-up described in Figure 4 was used without the addition of tracing powder. A high-speed camera recorded at a 250 fps frame rate and with a 45 μm/pixel resolution. An 8 MHz, 1-cycle, 2.5 mm of active diameter transducer was used. A spatiotemporal resolution of 187.5 μm × 8 ms with an overlap of 50% in temporal dimension was used. The Pulser-Receiver gain parameter was adjusted to 0 dB.

**Figure 11.**
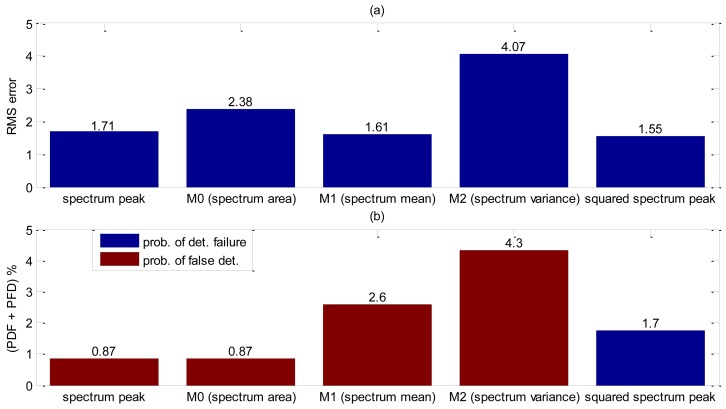
Accuracy results of detection liquid-gas interface without using tracing powder. (**a**) RMS error. (**b**) Probability of false detection and detection failure results.

Yet again, the shape parameter was investigated and each shape decision rule value obtained is listed in Table 3. By analyzing the values from Table 3, the first moment, M_1_, and the spectrum peak are very close and both deserve to be further investigated. Detection accuracy evaluation was carried on 115 bubbles, or 10 seconds of acquisition. Results obtained are summarized in Figure 11. Little difference is shown in RMS error value between M_1_ and spectrum peak, however M_1_ presents more than two times chance of bubble false detection. It is noteworthy that the accuracy results for this experiment were expected to be far better than the others because of the lack of particles.

### 3.3. Influence of Plexiglas Pipe

Plexiglas pipelines are used in many industrial plants and research rigs due to its resistance and transparency. These properties allow low to moderate operational pressure ranges while facilitating the use of optical techniques such as high speed filming.

**Figure 12.**
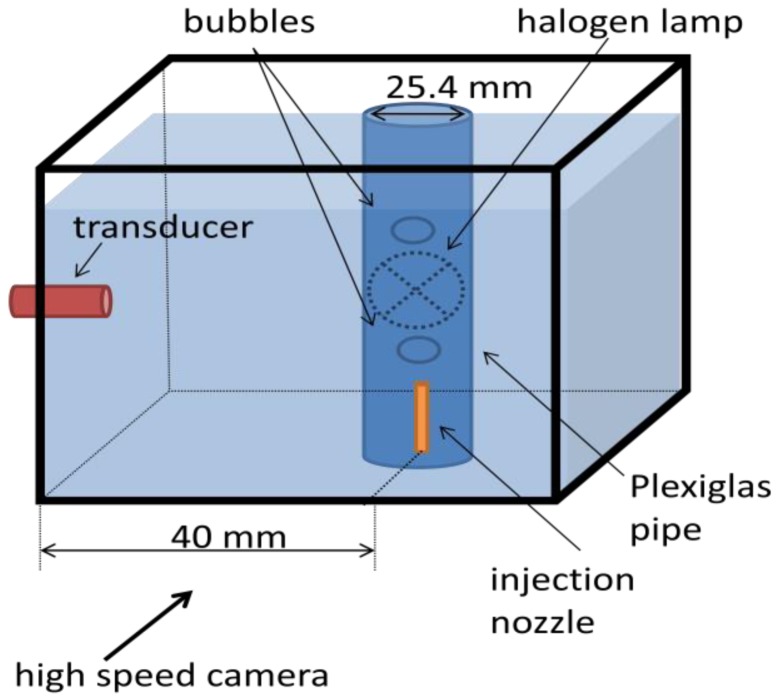
Experimental set-up for free-rising bubbles in quiescent liquid enclosed by a Plexiglas pipe.

Our objective is to discover whether the present technique would maintain a good performance in the presence of a pipe wall between a transducer and the flow. To simulate such a situation, a 25.4 mm ID Plexiglas pipe was used in an experimental configuration as shown in Figure 12.

The water tank was filled with mineral water. Seeding particles of 80 μm to 200 μm (EMS GRILTECH 1A P82), with 1.07 g/cm^3^ were added to the water to a concentration of 4 g/L. The high speed camera was set to a 250 fps frame rate and a 50 μm/pixel resolution. An 8 MHz, 1-cycle, 2.5 mm of active diameter transducer was used. Generated bubbles have a mean sphere-equivalent diameter of 3 mm. The measurement system was configured to spatiotemporal resolution of 187.5 μm × 8 ms with a 50% overlap in the temporal dimension. In this case it was necessary to adjust the Pulser-Receiver to a minimum gain of 40 dB.

As above, the shape parameter was investigated first. It was verified whether the previously obtained results would be maintained for this new experiment. The results presented in Table 4 show very little difference between shape parameter performances. These results must be carefully treated since the local deviation method gives only a rough estimation for the SNR value.

**Figure 13.**
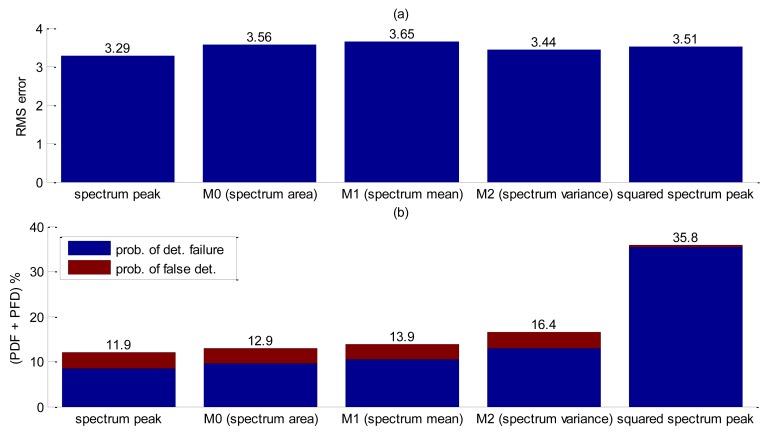
Accuracy performance results for selected shape parameters. (**a**) RMS error results. (**b**) Probability of false detection and detection failure results.

Therefore, RMS errors, detection failures and false detection probabilities were evaluated for each parameter in Table 4. A total number of 201 bubbles were released during this test. Edge detection was carried out by the Canny method. Analysis of Figure 13 shows that the spectrum peak presented the best performance for interface detection. Comparing these results with the free-rising flow without pipe interference (Figure 10), RMS error shows a slight increase. However, the combined probability (PDF + PFD) increases considerably thus corroborating that the pipe walls have a great influence on interface detection.

### 3.4. Influence of Metallic Wall

Industrial plants usually requires metallic pipes to support high pressure flows and for robustness. In this situation, the non-intrusive nature of ultrasound techniques is attractive. Nevertheless, ultrasound propagation through metal poses some difficulties. Because of acoustic impedance mismatch between metal and water, the coupling of ultrasound transducers to a metallic pipe may result in losses of signal energy. If the wall thickness to wave length ratio is different from *n*/2, with *n* = 1, 2, 3 …, a relatively strong reflection will occur which implicates in reduction of the transmission coefficient of the wave [20].

**Figure 14.**
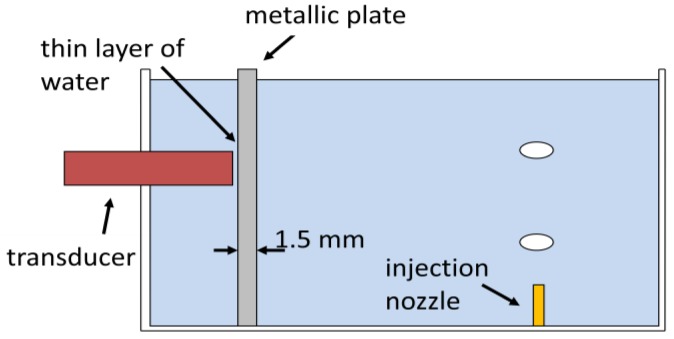
Cross-section diagram of metallic plate arrangement at the water tank.

An experiment using the same water tank with mineral water (without tracing powder) was set-up, but with a 1.5 mm thick, metallic plate (iron: sound velocity of 5900 m/s and acoustic impedance of 46.4 MRayls) obstructing the ultrasound transducer as shown in Figure 14. In this case it was necessary to adjust a minimum gain of 16 dB at Pulser-Receiver. Footage was done at 250 fps and with a 53 μm/pixel resolution. An 8 MHz, 1-cycle, 2.5 mm of active diameter transducer was used. And a 187.5 μm × 8 ms spatiotemporal resolution was with a 50% overlap in temporal dimension.

Since this is a very different set-up, a shape parameter investigation was carried out again, and results are summarized in Table 5.

**Figure 15.**
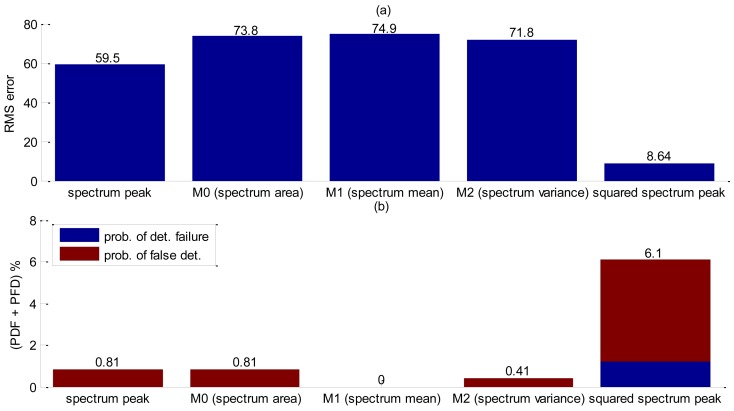
Accuracy results of interfacial detection with transducer obstructed by a metallic plate. (**a**) RMS error results. (**b**) Probability of false detection and detection failure results.

**Figure 16.**
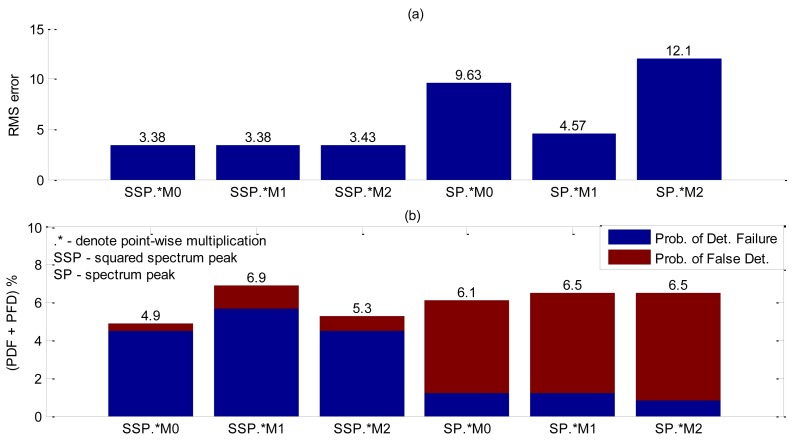
Accuracy results of interfacial detection with transducer obstructed by a metallic plate for shape parameters combined by point-wise multiplication. (**a**) RMS error results. (**b**) Probability of false detection and detection failure results.

For accuracy assessment a total of 246 bubbles releases were performed. The squared spectrum peak presented the bigger value (Table 5) and the best accuracy results as shown in Figure 15, but in this case it was further investigate whether the combination of two shape functions could lead to better accuracy results. Since squared spectrum peak surpassed others, it was combined, with each one of the five remaining shape parameters by means of a point-wise multiplication. Results obtained are summarized in Figure 16. The best arrangement was found to be the squared spectrum peak with zero moment (SSP.*M_0_). In Figure 16, the filtering of the squared spectrum peak by M_0_ shows a significant reduction in the RMS error compared to using only the squared spectrum peak (Figure 15). As for the probability of false detection, the SSP.*M_0_ shows a considerably reduction (Table 6) at the expense of an increasing detection failure. Nevertheless, comparing the total contribution of the two probabilities (PFD + PDF) the filtered one performs better (PFD + PDF = 4.9%) than the unfiltered (PFD + PDF = 6.1%). As spectrum peak presented a SDR value very close to the squared (Table 5), its accuracy has been also evaluated including the filtering by M_0_ at Figure 16 and Table 6. Using only spectrum peak it is not sufficient for the Canny edge to detect interface thus resulting in a high RMS error value (Figure 16).

This filtering shape was tested so as to determine whether it could work in any of the preceding experiments described in the previous section, but it only showed good results with metal coupling.

**Figure 17.**
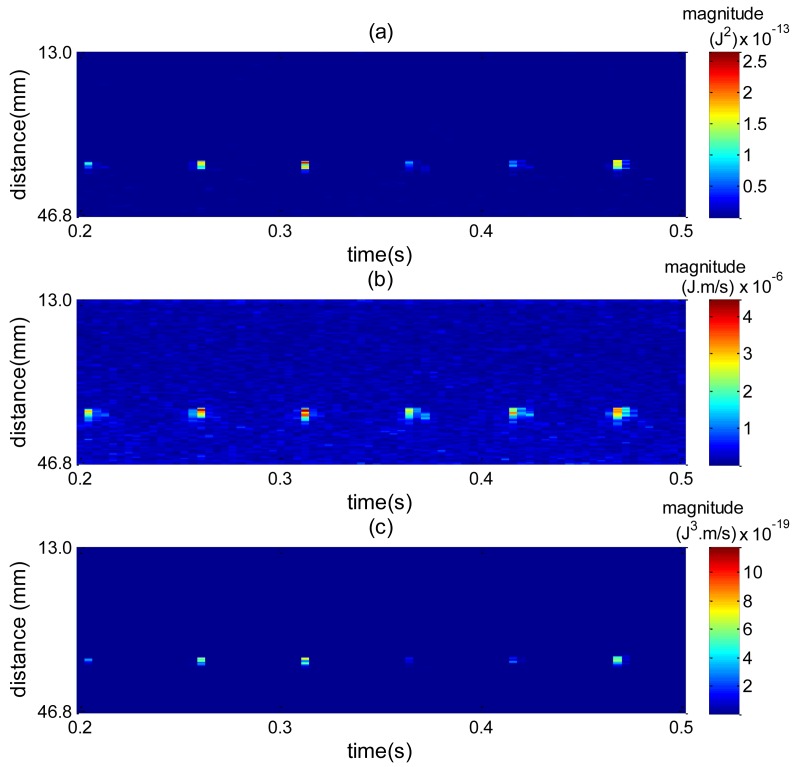
Comparison of spatiotemporal mapping for 0.3 s of acquisition. (**a**) Squared spectrum peak mapping. (**b**) Zero moment (M_0_) mapping. (**c**) Point-wise multiplication of squared spectrum peak and M_0_.

To understand the effect of combining squared spectrum peak and zero moment, Figure 17 plots a spatiotemporal map for each spectrum shape and then their combination. The high amplitude values are caused by bubble interface reflection. In Figure 17a high amplitude values are more spread around the bubble interface, which makes it difficult to reliably find edges. By combining Figure 17a with Figure 17b, Figure 17c is obtained, where the spread of values around the interface is considerably reduced. This way the edge detection can perform better, thus explaining the accuracy results obtained in Figure 16.

## 4. Conclusions

A velocity spectrum technique for detecting interfacial position in a free surface two phase flow that uses velocity spectrum data from VM-Spectrum algorithm was proposed.

We showed that it can be applied to free-rising bubbles flow with tracing particles in a quiescent liquid by using the spectrum first moment filtered by measured velocity. The method has exhibited RMS errors of 3.1% and 3.8% of combined probability of detection failure and false detection (PFD + PDF). When a transducer is mounted externally to a Plexiglas pipe, the VS technique performs better using the spectrum peak value, presenting little degradation in RMS error of 3.3 but showing more difficulty to detect bubbles as the probability increases by a factor of 3 (PFD + PDF = 11.9% ).

The methodology was further extended to the case where tracing powder was not used and, without particle echoes, it was unnecessary to filter the shape parameter map by velocity distribution (velocity filtering). For good accuracy, VS technique used the spectrum peak obtaining an RMS error of 1.71 and PDF + PFD of 0.89%. Finally we added a metallic plate to obstruct the transducer. In this case the best way to apply the VS technique is to use the squared peak spectrum value filtered by the zero spectrum moment obtaining a RMS error of 3.39 and PDF + PFD of 4.9%.

Although the technique was tested in a free surface flow it can be extended to pipe flow (there are some preliminary results that confirm this application). Like any ultrasound technique it is restricted to low void fractions, usually less than 20%, because of occlusion effect. Slug flow, which can reach a mean void fraction of 40%, is an exception of this restriction due to intermittent behavior. Powder concentration should be kept as low as possible because the superposition of backscattered echoes from a large quantity of tracer particles will generate high energy echoes which can be misinterpreted as a bubble.

## 5. Future Works

With the experience and knowledge obtained in this study, validation of the VS technique for two-phase pipe flow is an expected future endeavor. Analysis of velocity spectra from a large set of data shows that some of them can present more than one peak, usually due to noise or particles in liquid. Investigations of these secondary peaks in velocity spectrum could be carried out in order to suppress them, or to extract other shape parameters, like number of peaks, secondary peak amplitude and energy, in an attempt to maximize SNR and improve detection.

## Figures and Tables

**Table 1.** Results of shape parameters evaluation by SDR criterion.

**Shape Parameter**	**SDR**
spectrum peak	1.36
squared spectrum peak	1.42
M_0_ (spectrum area)	1.35
M_1_ (spectrum mean)	1.53
M_2_ (spectrum variance)	1.37

**Table 2.** List of tested sharpening filters. Only row gradient operators are shown, used column gradient operators were just transposed versions of the row array.

None	$\left[\begin{array}{ccc} 0 & 0 & 0\\ 0 & 1 & 0\\ 0 & 0 & 0 \end{array}\right]$	Frei-Chen	$\frac{1}{2+\sqrt{2}}\left[\begin{array}{ccc} 1 & 0 & -1\\ \sqrt{2} & 0 & -\sqrt{2}\\ 1 & 0 & -1 \end{array}\right]$
Sobel	$\frac{1}{4}\left[\begin{array}{ccc} 1 & 0 & -1\\ 2 & 0 & -2\\ 1 & 0 & -1 \end{array}\right]$	Roberts	$\left[\begin{array}{cc} 0 & -1\\ 1 & 0 \end{array}\right]$
Prewitt	$\frac{1}{3}\left[\begin{array}{ccc} 1 & 0 & -1\\ 1 & 0 & -1\\ 1 & 0 & -1 \end{array}\right]$	Separated pixel difference	$$\left[\begin{array}{ccc} 0 & 0 & 0\\ 1 & 0 & -1\\ 0 & 0 & 0 \end{array}\right]$$

**Table 3.** Results of shape parameters evaluation by SDR criterion for free-rising bubble flow without using tracing powder.

**Shape Parameter**	**SDR**
spectrum peak	0.97
squared spectrum peak	0.85
M_0_ (spectrum area)	0.78
M_1_ (spectrum mean)	0.99
M_2_ (spectrum variance)	0.72

**Table 4.** Results of shape parameters evaluation by SDR criterion for free-rising bubble flow encapsulated by a Plexiglas pipe.

**Shape Parameter**	**SDR**
spectrum peak	1.03
squared spectrum peak	1.05
M_0_ (spectrum area)	1.06
M_1_ (spectrum mean)	0.97
M_2_ (spectrum variance)	1.04

**Table 5.** Results of shape parameters evaluation by SDR criterion for free-rising bubble flow with a metallic plate obstructing the ultrasound transducer.

**Shape Parameter**	**SDR**
spectrum peak	1.82
squared spectrum peak	1.83
M_0_ (spectrum area)	1.23
M_1_ (spectrum mean)	1.70
M_2_ (spectrum variance)	1.08

**Table 6.** Accuracy results of interfacial detection with transducer obstructed by a metallic plate.

**Shape Parameter**	**RMS Error**	**PFD (%)**	**PDF(%)**
squared spectrum peak	8.64	4.9	1.2
(squared spectrum peak).*(M_0_)	3.39	0.4	4.5
spectrum peak	59.5	0	0.81
(spectrum peak).*(M_0_)	9.63	1.2	4.9
